# Comparison of dynamic susceptibility contrast enhanced MR and FDG-PET brain studies in patients with Alzheimer’s disease and amnestic mild cognitive impairment

**DOI:** 10.1186/s12967-022-03464-x

**Published:** 2022-06-07

**Authors:** Aleksandra Wabik, Elżbieta Trypka, Joanna Bladowska, Mikołaj Statkiewicz, Marek Sąsiadek, Anna Zimny

**Affiliations:** 1 Department of General and Interventional Radiology and Neuroradiology, Medical University Hospital, Borowska 213, 50-556 Wroclaw, Poland; 2 Department of Psychiatry, Wroclaw Medical University, Wroclaw, Poland; 3 Department of Radiology, Wroclaw Medical University, Wroclaw, Poland; 4 Affidea Imaging Center, Legnica, Poland

**Keywords:** Alzheimer’s disease, Amnestic mild cognitive impairment, Dynamic susceptibility contrast enhanced MRI, Fluorodeoxyglucose positron emission tomography, Cerebral perfusion, AD metabolic pattern

## Abstract

**Background:**

The aim of this study was to compare Dynamic Susceptibility Contrast Enhanced MRI (DSC-MRI) and PET with [18F]flurodeoxyglucose (FDG-PET) in the diagnosis of Alzheimer’s Disease (AD) and amnestic Mild Cognitive Impairment (aMCI).

**Methods:**

Twenty-seven age-and sex-matched patients with AD, 39 with aMCI and 16 controls underwent brain DSC-MRI followed by FDG-PET. Values of relative Cerebral Blood Volume (rCBV) and rCBV z-scores from frontal, temporal, parietal and PCG cortices were correlated with the rate of glucose metabolism from PET. Sensitivity, specificity and accuracy of DSC-MRI and FDG-PET in the diagnosis of AD and aMCI were assessed and compared.

**Results:**

In AD, hypoperfusion was found within all the examined locations, while in aMCI in both parietal and temporal cortices and left PCG. FDG-PET showed the greatest hypometabolism in parietal, temporal and left PCG regions in both AD and aMCI. FDG-PET was more accurate in distinguishing aMCI from the controls than DSC-MRI. In the AD and combined group (AD + aMCI) there were numerous correlations between DSC-MRI and FDG-PET results.

**Conclusions:**

In AD the patterns of hypoperfusion and glucose hypometabolism are similar, thus DSC-MRI may be a competitive method to FDG-PET. FDG-PET is a more accurate method in the diagnosis of aMCI.

## Background

Due to aging of the population, early diagnosis of dementia is an important problem in modern medicine. Alzheimer’s disease (AD) is a degenerative brain disease which accounts for 60–80% of dementia cases and is characterized by a decline in memory, language, problem-solving and other cognitive skills that affect a personʼs ability to perform everyday activities [1]. Many studies show that brain alterations in AD occur 20 or more years before the first clinical manifestations. The time between initial brain changes and the symptoms of advanced AD is known to represent the ‘continuum’ of Alzheimer’s pathology [1–3]. Amnestic mild cognitive impairment (aMCI) is considered a prodromal condition with a high risk of conversion to AD [1, 2].

For decades, the mechanism of neuronal degenerative changes leading to AD has been explained by amyloid cascade hypothesis. The accumulation of the β-amyloid protein outside neurons and deposition of an abnormal form of tau protein inside neurons is believed to contribute to the development of AD, leading to functional failure of synapses and structural damage to neurons [1]. Recently, in addition to amyloid, a vascular hypothesis has been postulated. On the one hand, it assumes that the amyloid itself shows both neuronal and endothelial toxicity leading to brain degeneration and hypoperfusion [4]. On the other hand, cerebrovascular risk factors, including a higher level of high-sensitivity C-Reactive Protein (hsCRP) and lower level of high-density lipoprotein (HDL), may cause disturbances of macro- or micro-vasculature circulation and endothelial damage that contributes to amyloid accumulation and to neuronal death [5, 6]. The vascular hypothesis is also consistent with the new concept of the neurovascular unit, stating that not only is amyloid angiotoxic, but chronic hypoperfusion and vascular damage may further accumulate β-amyloid and exacerbate brain degeneration [7].

A modern diagnosis of AD, apart from clinical assessment, requires also the incorporation of other biomarkers. The National Institute on Aging (NIA) and the Alzheimer’s Association identified two categories: biomarkers showing the level of β-amyloid accumulation in the brain (assessed with amyloid PET) and low levels of β42-amyloid in cerebral spinal fluid, as well as biomarkers showing injury or degeneration of the brain neurons measured with high levels of tau in cerebral spinal fluid, brain atrophy determined with anatomic magnetic resonance imaging (MRI) and brain hypometabolism assessed by [18F]fluorodeoxyglucose-Positron Emission Tomography (FDG-PET) [3].

Structural MRI plays an important role in the diagnosis of dementia, firstly in excluding secondary causes of cognitive impairment such as vascular lesions, brain tumors or hydrocephalus, and secondly in the assessment of the distribution of brain atrophy. A typical pattern of brain atrophy in the course of AD degeneration involves medial temporal lobes and temporo-parietal areas including posterior cingulate gyrus (PCG), followed by frontal lobe atrophy in advanced cases [8, 9]. Moreover, modern advanced MR techniques allow for the assessment of not only brain structure, but also its function or metabolism. One such method is Dynamic Susceptibility Contrast Enhanced MRI (DSC-MRI), enabling an insight into the cerebral microcirculation on the basis of evaluation of the first pass of a contrast material through the brain microvasculature. The method requires intravenous injection of a paramagnetic contrast material containing gadolinium chelates that distorts the magnetic field and induces signal loss. The decrease in signal intensity as the contrast medium passes through the vascular bed is illustrated by the perfusion curve. It allows calculation of several quantitative hemodynamic parameters such as: cerebral blood volume (CBV), cerebral blood flow (CBF), mean transit time (MTT), and time to peak (TTP). Cerebral blood volume (CBV) is one of the most important perfusion parameters, which is defined as the volume of blood in a given amount of brain tissue (ml of blood per 100 g of brain tissue) thus showing the regional blood supply of the brain tissue. This parameter has been widely analyzed in many previous publications on different brain pathologies, including brain tumors, cerebral inflammation or demyelination [10, 11]. There have been only a few reports on DSC-MR perfusion in dementia and they also looked at the parameter of CBV [12–18]. They have shown a significant reduction in CBV values in the temporo-parietal cortex, including PCG with a relative sparing of the sensorimotor cortex in AD [12, 13, 15, 16] while in aMCI, hypoperfusion was reported mainly in PCG [17, 18]. Some studies have shown a significant correlation of perfusion results with neuropsychological tests in AD and MCI [16, 17].

PET represents a diagnostic nuclear medicine modality that assesses pathophysiologic and chemical processes by using radiopharmaceuticals that mimic endogenous molecules. [18F]FDG is the most common molecular imaging biomarker used in PET. In particular, [18F]FDG is a radio-labeled glucose analogue and thus by entering the glucose metabolic pathway, provides information about tissue metabolism. It has a wide variety of applications in neurosciences, oncology, cardiology and also in dementia [19, 20]. FDG-PET evaluates the regional cerebral metabolic rate of glucose, thus giving information about the entity of neuronal loss or synapse dysfunction and the reduced brain glucose metabolism is associated with neurodegenerative diseases. In AD this examination shows glucose hypometabolism in very specific locations, called the “AD metabolic patternˮ including temporo-parietal associative cortex, PCG, precuneus, medial temporal lobes, especially in the entorhinal cortex and the hippocampus [19–24]. In the advanced course of the disease changes occur in the frontal cortex, with saving the primary sensorimotor cortex. However, in MCI, decreased metabolism is mainly found in PCG, and to a lesser extent in the temporo-parietal area, which may be a sensitive prognostic indicator of conversion to AD [23, 25–28]. Even though FDG-PET is a great method in the evaluation of early changes in the brain of AD patients or even in predementia states such as aMCI, it requires injection of a radionuclide tracer and uses ionizing radiation, since it is performed in conjunction with CT (PET/CT scanner). Moreover, in some countries the use of PET-CT in everyday clinical practice is limited due to high costs and limited availability.

Looking at previous studies, structural, perfusion and metabolic alterations in AD or aMCI seem to follow the same patterns but there are not many reports directly comparing different imaging techniques within the same groups of patients. Perfusion and metabolic changes have been reported to precede structural atrophy but there have been only a few reports focusing on the comparison of DSC-MR perfusion with the results of FDG-PET studies in AD and aMCI [15, 16]. In the first paper by Gonzales et al. the authors performed their study on patients with AD, using only visual evaluation of rCBV maps derived from DSC-MR perfusion and brain glucose metabolism maps from FDG-PET studies [15]. In the second report, Zimny et al. looked for correlations between DSC-MR perfusion and FDG-PET results in MCI subjects but only focusing on one brain area that was PCG without evaluation of other cortical regions [18]. For this reason, our study comparing DSC-MR perfusion with FDG-PET results in AD and aMCI fills the gap in the existing scientific literature.

The aim of our study was to establish the role of DSC-MR perfusion in relation to FDG-PET imaging based on a detailed comparison of these two techniques. The main assumption of our research was that DSC-MR perfusion results should be similar to FDG-PET studies because the glucose metabolism is partially dependent on cerebral perfusion. The comparison of DSC-MR perfusion and FDG-PET was performed based on: (1) the assessment of hypoperfusion and hypometabolism patterns in the selected brain areas in AD and aMCI, (2) the assessment of correlations between the results of these two techniques and analysis of their accuracy in diagnosis of AD and aMCI, (3) the assessment of correlation between the results of DSC-MR perfusion or FDG-PET and the severity of cognitive impairment in AD and aMCI.

We hypothesized that DSC-MR perfusion study could be competitive with the FDG-PET examination but with several advantages, such as no ionizing radiation, better availability and lower costs.

## Methods

### Subjects

The research material consisted of 66 patients: 27 with AD (mean age 70.33 years, mean MMSE 18.67 points), 39 diagnosed with aMCI (mean age 66.6 years, mean MMSE 26.2 points). In addition, a control group of 16 subjects (mean age 65 years, mean MMSE 27.1 points) was recruited. All subjects underwent detailed psychiatric examination, as well as laboratory and neuropsychological tests, including Mini-Mental State Examination (MMSE) adjusted for age and education level, Clinical Dementia Rating (CDR), Clock Drawing Test, Test Your Memory, Dementia Toolkit for Effective Communication, verbal fluency FAS test, Instrumental Activity of Daily Living, and Geriatric Depression Scale. The distribution of age, gender and MMSE scores for each group are presented in Table 1. The study was conducted in accordance with the guidelines of the University Ethics Committee for conducting research involving humans. Each patient provided his/her signed consent to participate in the study.

**Table 1:** The distribution of age, gender, MMSE scores within the subject groups (mean ± standard deviation)

	AD	aMCI	CG
Patients (n)	27	39	16
Age (years)	70.33 ± 8.68	66.59 ± 10.2	65 ± 8.38
Gender (male/female)	10/17	20/19	4/12
MMSE (points)	18.67 ± 5	26.2 ± 1.87	27.1 ± 1.2

*AD* Alzheimer’s disease; *aMCI* amnestic mild cognitive impairment; *CG* control group, *MMSE* Mini-Mental State Examination (severe dementia 0–10 points, moderate dementia 11–18 points, mild dementia 19–23 points, mild cognitive impairment without dementia 24–26 points, normal 27–30 points)

### Magnetic resonance examination

All MR examinations of the brain were performed with a 1.5 Tesla MR scanner (Signa Hdx, GE Medical Systems) using a 16-channel HNS (head-neck-spine) coil. Standard structural protocol was followed by DSC-MR perfusion using fast echo planar (EPI) gradient.

**Fig. 1:**
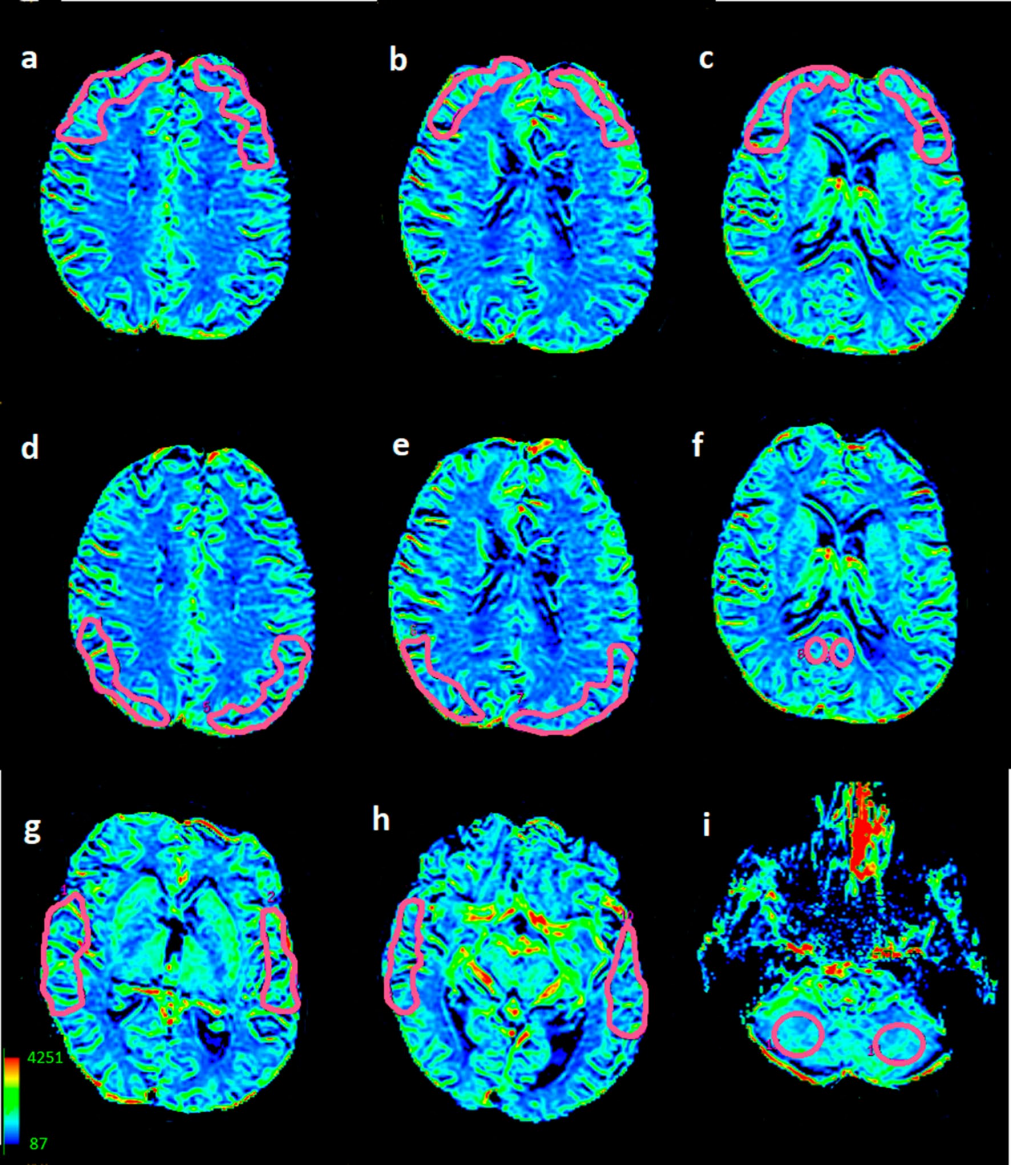
Location of Regions of Interest on CBV maps derived from DSC-MR perfusion in a single subject. Right and left frontal cortices (**a**–**c**), right and left parietal cortices (**d**, **e**), right and left posterior aspects of the cingulate gyri (PCG) (**f**), right and left temporal cortices (**g**, **h**), right and left cerebellar hemispheres (**i**)

T2*-weighted sequences with the following parameters: TR = 1.900 ms, TE = 80 ms, FOV = 30 cm, matrix = 192 × 128, slice thickness = 8 mm without spacing, NEX—1.0. Ten seconds after the start of image acquisition, a bolus of 1.0 mol/l gadobutrol formula (Gadovist, Schering, Berlin, Germany) in a dose of 0.2 ml/kg of body weight was injected via a 20-gauge catheter placed in the antecubital vein. Contrast administration was performed using an automatic injector (Medrad) at a rate of 5 ml/s and was followed by a saline bolus (20 ml at 5 ml/s). Perfusion imaging lasted 1 min 26 s, in which sets of images from 13 axial slices were obtained before, during, and after contrast injection. The dynamic images were post-processed into parametric perfusion maps using Functool software (GE, ADW 4.6). Maps of Cerebral Blood Volume (CBV) were computed on a pixel-wise basis from the first-pass data from the capillary bed. Values of CBV were obtained using manually drawn Regions of Interest (ROI) bilaterally within the frontal, temporal and parietal cerebral cortex (500–900 mm^2^ in size), and within the posterior aspect of the cingulate gyrus (100–200 mm^2^ in size). To cover bigger cortical areas, the ROIs in the frontal lobes were drawn on three adjacent scans, while in the parietal and temporal lobes on two adjacent scans, and then the CBV values obtained from these ROIs were mathematically averaged to one frontal, temporal or parietal cortical value, separately for the right and left hemisphere. All CBV values were normalized to the mean CBV value of the cerebellar cortex in order to obtain the relative CBV (rCBV). The cerebellar cortex was chosen as the reference area because it is the region less affected in AD compared to other cortical measures [23]. The ROI in the cerebellum was approximately 300–400 mm^2^ in size (Fig. 1). The location of the ROIs was chosen to best correspond with the glucose metabolism measurements in the FDG-PET study (Table 2).

**Table 2:** Nomenclature of the analyzed brain areas

Cortical regions in FDG-PET provided by CORTEX ID software	Cortical regions analysed in DSC-MRI using manual ROI placement	Unified names of the analyzed cortical regions used in the study
Frontal Association Right	Mean of the three ROIs from the right frontal cortex	R frontal (right frontal cortex)
Frontal Association Left	Mean of the three ROIs from the left frontal cortex	L frontal (left frontal cortex)
Temporal Association Right	Mean of the two ROIs from the right temporal cortex	R temporal (right temporal cortex)
Temporal Association Left	Mean of the two ROIs from the left temporal cortex	L temporal (left temporal cortex)
Parietal Association Right	Mean of the two ROIs from the right parietal cortex	R parietal (right parietal cortex)
Parietal Association Left	Mean of the two ROIs from the left parietal cortex	L parietal (left parietal cortex)
Posterior Cingulate Right	One ROI in the posterior part of the right cingulate gyrus	R PCG (right posterior cingulate gyrus)
Posterior Cingulate Left	One ROI in the posterior part of the left cingulate gyrus	L PCG (left posterior cingulate gyrus)

*ROI* region of interest, *DSC-MRI* dynamic susceptibility contrast enhanced magnetic resonance imaging, *FDG-PET* fluorodeoxyglucose positron emission tomography

### PET examination

**Fig. 2:**
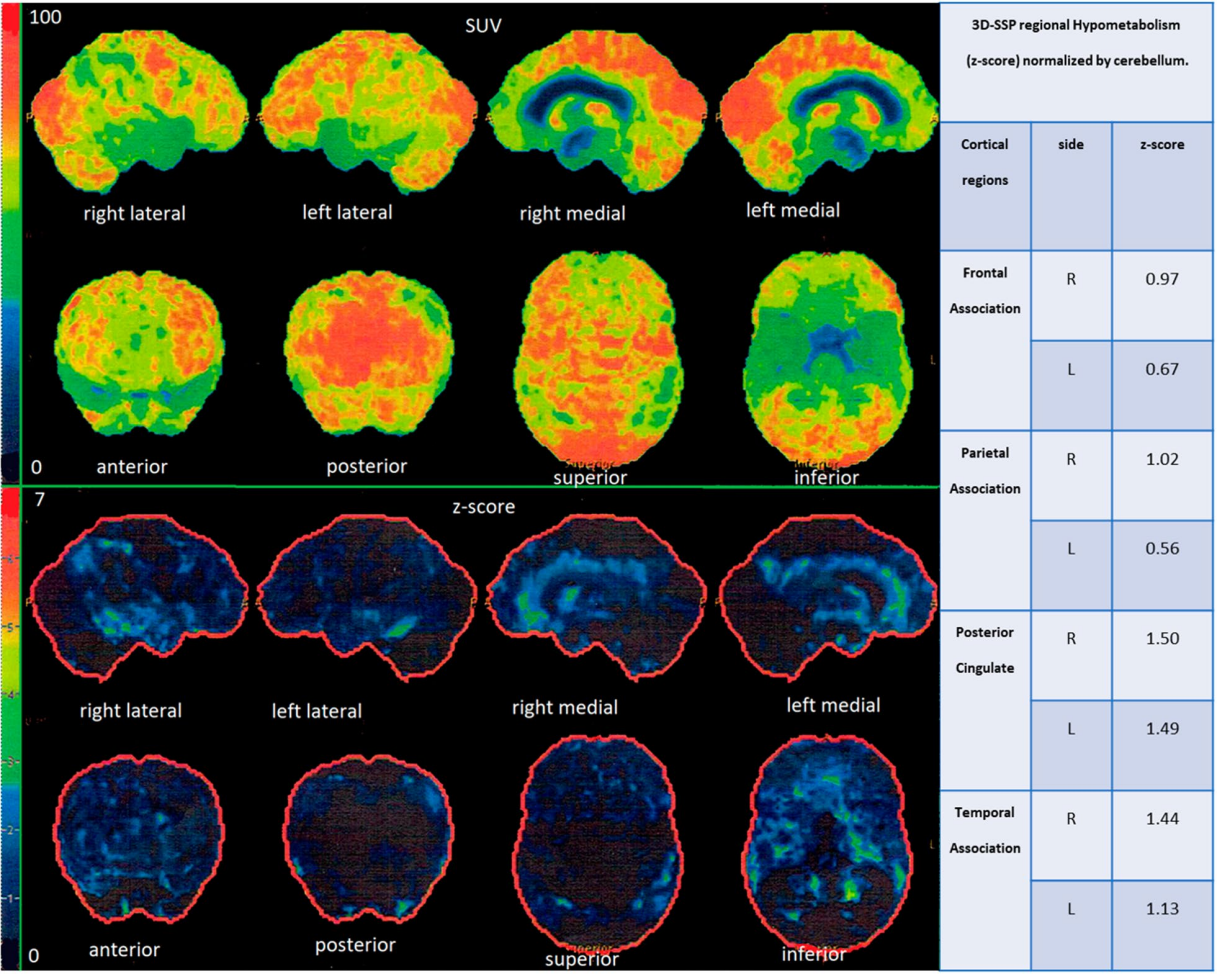
Results of the FDG-PET study in single patient generated automatically by the CORTEX ID software. Standard Uptake Value (SUV) and z-score maps presented as three-dimensional Stereotactic Surface Projection (3D-SSP) images of the brain cortex and a table of parametrical values of z-scores for different cortical locations. SUV maps show an absolute glucose metabolism with the red color indicating a high glucose metabolism (hypermetabolism), green color a normal glucose metabolism and in blue color a decreased glucose metabolism (hypometablism). Z-score maps show a cerebral glucose metabolism in relation to the rate of the glucose metabolism of the control group; on z-score maps red color indicates severe hypometabolism, green intermediate hypometabolism and blue color a normal glucose metabolism

PET studies were performed within 3 weeks after MR examination. The PET images were obtained using a GE Discovery STE16 PET/CT scanner with [18F]fluorodeoxyglucose (FDG) as a radiotracer. All participants fasted for at least 6 h before examination. Data acquisition lasted 8 min and was performed 30 min after intravenous injection of 5 MBq/kg of FDG. Detector spatial resolution was 5.6 mm and data were displayed on a 128 × 128 pixel matrix. To avoid external stimulation during FDG uptake, patients stayed in a resting condition in a darkened room. The acquired data were processed using iterative reconstructions. Attenuation and scatter corrections were made simultaneously by transmission measurements using CT. Next, PET/CT images were transferred to a workstation (GE Healthcare) and processed using a commercial CORTEX ID application. Scans were spatially normalized to a stereotactic space based on the Talairach and Tournoux atlas [29]. Then brain images underwent size correction to standard dimensions of 3D-atlas and a regional anatomic variants correction to decrease individual variations. All data were normalized to the mean FDG uptake value of the cerebellum, where glucose utilization is comparatively preserved in dementia [30]. Realigned FDG-PET scans of all subjects were compared with a normative, age stratified reference database included in the CORTEX ID software. Glucose metabolic activity was automatically determined in 14 cerebral regions as Standard Uptake Values (SUV) followed by automatic calculations of glucose metabolism z-scores. In each subject the FDG-PET results were presented as maps of glucose metabolism (SUV maps), maps of z-scores and a table with the numerical results of z-scores for different cortical locations (frontal, temporal, parietal and PCG) (Fig. 2).

### Statistical analysis

To compare DSC-MRI with the FDG-PET studies, two types of perfusion parameters were used, such as rCBV values and rCBV z-score. The rCBV z-score was used to make the MR results as similar as possible to the FDG-PET results, in which the level of glucose metabolism is automatically presented in the form of a z-score that indicates the number of standard deviations (SD) of a given parameter from a population norm. To calculate CBV z-scores from the DSC-MR perfusion study we used a mathematical formula as follows: [(mean CBV of the control group − mean CBV of a subject)/SD of the control group]. In DSC-MRI higher z-scores indicated higher rates of perfusion impairment (the higher the z-score the more severe the hypoperfusion) whereas in FDG-PET higher z-scores meant higher rates of metabolic impairment (the higher the z-score the more pronounced the glucose hypometabolism).

The comparisons of mean age and the results of DSC-MRI and FDG-PET between the AD, MCI and CG groups were carried out using the ANOVA method followed by a Scheffe’s post-hoc test to compare the results in pairs between MCI and CG, AD and CG, as well as AD and MCI. Analyzes of correlation between DSC-MR perfusion and FDG-PET results, as well as between the results of imaging studies (both MR and PET) and the results of psychological tests were performed using the Pearson correlation coefficient.

Additionally, the sensitivity and specificity of MR and PET parameters in differentiating between AD, MCI and CG were calculated using the Receiver-Operating Characteristic (ROC) method, in which the accuracy of the test is indicated by the area under the ROC curve. In all statistical analyzes, a p value of  < 0.05 was considered statistically significant. In the case of the rCBV z-score and PET z-score parameters, z-scores  ≥ 1 were considered to be significantly different from the CG.

## Results

In AD patients compared to CG, DSC-MRI results showed significantly decreased rCBV values (p ≤ 0.05) and significantly higher z-score rCBV values (z-score ≥ 1) in all the examined cortical locations.

Compared to healthy controls, MCI patients showed a significant decrease of rCBV values within the cortex of both parietal and temporal lobes and left PCG, while by using the rCBV z-score, significant hypoperfusion was found within the right parietal lobe.

The AD group, when compared to the MCI group, showed significantly lower rCBV values and higher rCBV z-scores within all the examined areas of the brain cortex (Table 3).

**Table 3:** Comparison of MR perfusion and FDG-PET results from different cortical locations between AD, MCI and control groups

Cortical location	Mean rCBV							rCBV z-score			PET z-score		
	Study groups			ANOVA	Sheffeʼs post-hoc test			Study groups		Sheffeʼs post-hoc test	Study groups		Sheffeʼs post-hoc test
	AD (SD)	MCI (SD)	CG (SD)	p value	AD vs CG p value	MCI vs CG p value	AD vs MCI p value	AD (SD)	MCI (SD)	AD vs MCI p value	AD (SD)	MCI (SD)	AD vs MCI p value
R frontal	0.90 (0.11)	1.09 (0.19)	1.18 (0.17)	< 0.001*	< 0.001*	0.22	< 0.001*	1.64 (0.66)	0.51 (1.55)	< 0.001*	1.55 (1)	0.9 (0.44)	< 0.001*
L frontal	0.86 (0.1)	1.09 (0.22)	1.18 (0.13)	< 0.001*	< 0.001*	0.26	< 0.001*	2.44 (0.83)	0.65 (1.66)	< 0.001*	1.93 (1.25)	0.97 (0.49)	< 0.001*
R temporal	0.85 (0.09)	1.11 (0.18)	1.29 (0.3)	< 0.001*	< 0.001*	0.013*	< 0.001*	1.4 (0.29)	0.57 (0.61)	< 0.001*	2.24 (0.86)	1.02 (0.4)	< 0.001*
L temporal	0.85 (0.1)	1.09 (0.17)	1.3 (0.4)	< 0.001*	< 0.001*	0.01*	< 0.001*	1.12 (0.24)	0.52 (0.43)	< 0.001*	2.52 (0.96)	1.01 (0.47)	< 0.001*
R parietal	0.83 (0.09)	1.08 (0.18)	1.23 (0.14)	< 0.001*	< 0.001*	0.005*	< 0.001*	2.9 (0.68)	1.08 (1.33)	< 0.001*	2.5 (1.1)	1.31 (0.58)	< 0.001*
L parietal	0.81 (0.10)	1.08 (0.20)	1.24 (0.16)	< 0.001*	< 0.001*	0.008*	< 0.001*	2.64 (0.6)	0.95 (1.2)	< 0.001*	2.73 (1.23)	1.29 (0.61)	< 0.001*
R PCG	0.8 (0.11)	1.06 (0.22)	1.2 (0.22)	< 0.001*	< 0.001*	0.056	< 0.001*	1.83 (0.53)	0.64 (1.02)	< 0.001*	1.84 (0.71)	0.94 (0.58)	< 0.001*
L PCG	0.79 (0.11)	1.09 (0.22)	1.24 (0.23)	< 0.001*	< 0.001*	0.040*	< 0.001*	1.96 (0.48)	0.64 (0.94)	< 0.001*	2.12 (0.73)	1.03 (0.55)	< 0.001*

*SD* standard deviation, *R* right, *L* left, *PCG* posterior cingulate gyrus, *AD* Alzheimer’s disease, *MCI* mild cognitive impairment, *CG* control group, *rCBV* relative cerebral blood volume

*Statistically significant values, p < 0.05

**Fig. 3:**
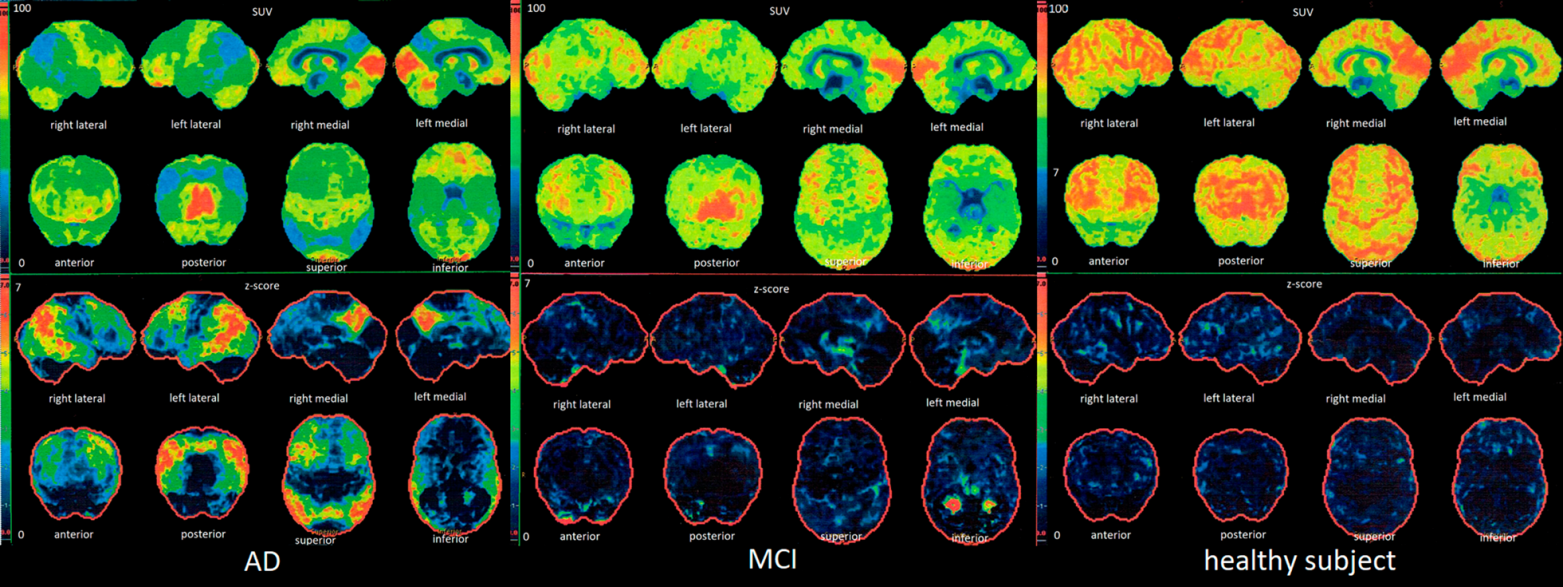
The results of FDG-PET studies in subjects with AD, MCI and a healthy person generated automatically by the CORTEX ID software. Different rates of glucose metabolism: the most severe hypometabolism in AD (visible in PCG and temporo-parietal cortices), intermediate hypometabolism in MCI (only in the PCG region) and normal in the control group (no hypometabolism is visible)

The FDG-PET study in the AD group showed significant glucose hypometabolism within all measured areas of the cerebral cortex, while in the MCI group within the cortex of both parietal and temporal regions and left PCG. The greatest impairment of glucose metabolism in patients with AD, as well as in patients with MCI, was demonstrated in the parietal, temporal and left PCG regions. The AD patients compared to MCI subjects showed significantly higher impairment of glucose metabolism in all evaluated locations (Table 3). The third figure shows results of the FDG-PET studies in exemplary subjects with AD, MCI and a healthy person generated automatically by the CORTEX ID software (Fig. 3).

**Fig. 4:**
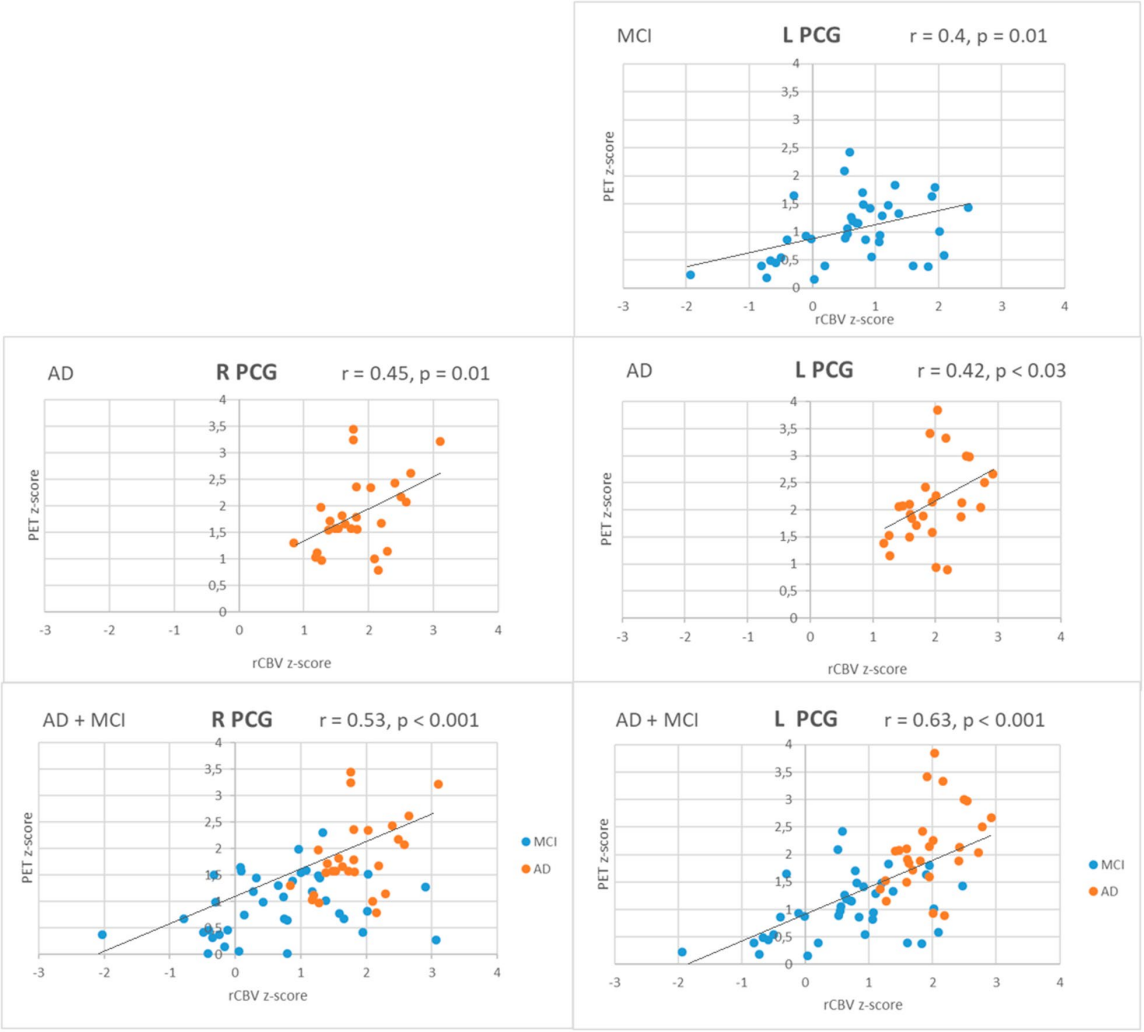
Graphs showing the most significant correlations between rCBV z-scores and PET z-scores in PCG. *L PCG* left posterior cingulate gyrus, *R PCG* right posterior cingulate gyrus, *AD* Alzheimer’s disease, *MCI* mild cognitive impairment, *rCBV* relative cerebral blood volume, *r* Pearson correlation coefficient, *p* probability value

In AD patients statistically significant positive correlations between MR perfusion and FDG-PET results were found for almost all the evaluated cortical regions, apart from the right parietal cortex. In the MCI group there was only one single correlation between these two techniques found within the left PCG (r = 0.4, p = 0.01). In the combined group (AD + MCI) the PET z-score and rCBV z-score analysis showed statistically significant positive correlations in all locations. These correlations were strongly positive in the area of PCG and in the temporal lobes (r > 0.5), moderately positive in the area of the parietal lobes, and weaker in other locations (r < 0.5) (Table 4). The fourth figure shows exemplary graphs of the correlation between rCBV z-score and PET z-score in the right and left PCG regions in AD, MCI and the combined group (Fig. 4).

**Table 4:** The results of correlation between rCBV z-score and FDG-PET z-score separately for AD and MCI groups and for all patients (AD + MCI)

Location	AD	MCI	AD + MCI
R front	r = 0.47 p = 0.01*	r =− 0.12 p = 0.46	r = 0.30 p = 0.01*
L front	r = 0.39 p = 0.04*	r =− 0.16 p = 0.31	r = 0.31 p = 0.01*
R temp	r = 0.49 p = 0.01*	r = 0.27 p = 1	r = 0.6 p < 0.001*
L temp	r = 0.48 p = 0.01*	r = 0.02 p = 0.88	r = 0.55 p < 0.001*
R pariet	r = 0.25 p = 0.2	r = 0.15 p = 0.35	r = 0.46 p < 0.001*
L pariet	r = 0.5 p = 0.007*	r =− 0.1 p = 0.55	r = 0.45 p < 0.001*
R PCG	r = 0.45 p = 0.01*	r = 0.28 p = 0.8	r = 0.53 p < 0.001*
L PCG	r = 0.42 p = 0.03*	r = 0.4 p = 0.01	r = 0.63 p < 0.001*

*r* Pearson correlation coefficient, *p* probability value, *SD* standard deviation, *R* right, *L* left, *PCG* posterior cingulate gyrus, *AD* Alzheimer’s disease, *MCI* mild cognitive impairment

*Statistically significant values, p < 0.05

In distinguishing MCI from CG, the highest sensitivity, specificity and accuracy (0.95, 1.0 and 0.95, respectively) were found for the PET z-score, followed by rCBV z-score and rCBV (Table 5). The highest sensitivity, specificity and accuracy (0.98, 1.0 and 0.98, respectively) in distinguishing AD from CG were revealed for the rCBV z-score, followed by FDG-PET z-score and rCBV (Table 5). Lastly, in differentiating AD from MCI, the same sensitivity, specificity and accuracy (0.66, 0.94 and 0.84, respectively) were found for both rCBV z-score and rCBV (Table 5).

**Table 5:** Average values of cut off, sensitivity, specificity and accuracy of DSC-MRI and FDG-PET studies in the diagnosis of AD and MCI

Compared groups	Parameter	Cut-off	Sensitivity	Specificity	Accuracy
MCI vs CG	rCBV	1.1	0.57	0.80	0.68
	rCBV z-score	0	0.76	1.00	0.77
	PET z-score	0	0.95	1.00	0.95
AD vs CG	rCBV	1.01	0.94	0.93	0.95
	rCBV z-score	0	0.98	1.00	0.98
	PET z-score	0	0.97	1.00	0.97
AD vs MCI	rCBV	1.01	0.66	0.94	0.84
	rCBV z-score	1.13	0.66	0.94	0.84
	PET z-score	1.34	0.82	0.77	0.81

*AD* Alzheimer’s disease, *MCI* mild cognitive impairment, *rCBV* relative cerebral blood volume

The results of the study did not reveal many statistically significant correlations between FDG-PET or DSC-MR perfusion parameters and the results of the MMSE test in the separate MCI and AD groups. In AD, statistically significant correlations (r = 0.3–0.4) were found with the results of MR perfusion from the left parietal and left temporal lobes. When analyzing patients with MCI, statistically significant correlations were shown (r = 0.3–0.4) for PCG and both parietal cortices. In turn, in the FDG-PET study, statistically significant correlations (r = 0.47) with MMSE were found only in AD patients with the results from the left frontal lobe. After combing all AD and MCI subjects in one bigger group (AD + MCI), a statistically significant correlation between MR perfusion or FDG-PET results and MMSE test was found in all the examined locations (Table 6).

**Table 6:** The results of correlation between MR perfusion, FDG-PET and Mini-Mental test (MMSE)

Cortical location	AD			MCI			AD + MCI		
	rCBV	rCBV z-score	PET z-score	rCBV	rCBV z-score	PET z-score	rCBV	rCBV z-score	PET z-score
R frontal	r = 0.16 p = 0.43	r =**− **0.16 p = 0.43	r =**− **0.28 p = 0.14	r = 0.22 p = 0.18	r =**− **0.22 p = 0.18	r = 0.21 p = 0.198	r = 0.46 p < 0.001*	r =− 0.46 p < 0.001*	r =− 0.42 p < 0.001*
L frontal	r = 0.2 p = 0.29	r =**− **0.2 p = 0.29	r =− 0.47 p = 0.014*	r = 0.29 p = 0.073	r =**− **0.29 p = 0.073	r = 0.025 p = 0.12	r = 0.52 p < 0.001*	r =− 0.52 p < 0.001*	r =− 0.56 p < 0.001*
R temporal	r = 0.28 p = 0.16	r =**− **0.28 p = 0.16	r = 0.16 p = 0.4	r = 0.18 p = 0.26	r =**− **0.18 p = 0.26	r =**− **0.03 p = 0.85	r = 0.57 p < 0.001*	r =− 0.57 p < 0.001*	r =− 0.47 p < 0.001*
L temporal	r = 0.4 p = 0.03*	r =− 0.4 p = 0.03*	r =**− **0.25 p = 0.21	r = 0.07 p = 0.69	r =**− **0.07 p = 0.69	r =**− **0.04 p = 0.77	r = 0.57 p < 0.001*	r =− 0.57 p < 0.001*	r =− 0.65 p < 0.001*
R parietal	r = 0.11 p = 0.57	r =**− **0.11 p = 0.57	r =**− **0.01 p = 0.96	r = 0.34 p = 0.034*	r =− 0.34 p = 0.034*	r = 0.05 p = 0.74	r = 0.57 p < 0.001*	r =− 0.57 p < 0.001*	r =− 0.43 p < 0.001*
L parietal	r = 0.38 p = 0.048*	r =− 0.38 p = 0.048*	r =**− **0.36 p = 0.06	r = 0.34 p = 0.035*	r =− 0.34 p = 0.035*	r = 0.52 p = 0.75	r = 0.62 p < 0.001*	r =− 0.62 p < 0.001*	r =− 0.61 p < 0.001*
R PCG	r =**− **0.05 p = 0.8	r =**− **0.05 p = 0.8	r =**− **0.14 p = 0.49	r = 0.4 p = 0.009*	r =− 0.4 p = 0.009*	r =**− **0.5 p = 0.74	r = 0.5 p < 0.001*	r =− 0.5 p < 0.001*	r =− 0.4 p = 0.001*
L PCG	r = 0.11 p = 0.56	r =**− **0.11 p = 0.56	r =**− **0.17 p = 0.38	r = 0.37 p = 0.024*	r =− 0.37 p = 0.024*	r =**− **0.14 p = 0.49	r = 0.58 p < 0.001*	r =− 0.58 p < 0.001*	r =− 0.57 p < 0.001*

*r* Pearson correlation coefficient, *R* right, *L* left, *PCG* posterior cingulate gyrus, *AD* Alzheimer’s disease, *MCI* mild cognitive impairment, *rCBV* relative cerebral blood volume

^*^Statistically significant values, p < 0.05

## Discussion

The aim of our study was to compare DSC-MR perfusion and FDG-PET studies based on the assessment of: (1) hypoperfusion and hypometabolism patterns in the selected brain areas in AD and MCI, (2) correlation between the results of these two techniques and their accuracy in diagnosis of AD and MCI, and (3) correlation between the results of DSC-MRI and FDG-PET with the severity of cognitive impairment in AD and MCI.

In our study AD patients, compared to the control group, showed significant hypoperfusion in all examined cortical locations. Our results are consistent with the typical pattern of Alzheimerʼs degeneration and hypoperfusion reported in numerous publications within the PCG, temporo-parietal cortices, and in later stages also frontal cortices with relative sparing of the sensorimotor cortex [12, 13, 15, 31, 32]. In the MCI group, compared to controls, we found significantly decreased rCBV values within the cortex of both parietal lobes, temporal lobes and left PCG, while by using the rCBV z-scores, significant hypoperfusion was detected in the right parietal cortex, which is also consistent with the pattern of very early alterations in the course of AD pathology [13, 17, 18, 32, 33]. In the MCI group perfusion alterations were less severe than in the AD group, which supports the theory that hypoperfusion is a marker of neuronal damage and becomes more prominent in the later stages of AD.

In our study the FDG-PET results in the AD group showed significant glucose hypometabolism in all investigated locations of the cerebral cortex reported before [24] and the most pronounced in the parietal, temporal and left PCG regions, followed by hypometabolism in the frontal cortices. These results are in accordance with the commonly accepted metabolic pattern in the course of AD, thanks to which, as demonstrated by Mosconi et al. it is possible to differentiate AD from DLB (Dementia with Lewy bodies) and FTLD (frontotemporal lobar degeneration) even in the advanced forms [20, 22]. MCI subjects showed less severe hypometabolism mainly in the parieto-temporal regions and left PCG, which is in line with the existing literature [23, 24]. Mosconi et al. suggests that FDG-PET is a good diagnostic method in detecting the early stages of dementia already at the MCI level. In her work, the typical AD pattern of glucose hypometabolism was observed in 79% of MCI subjects with deficits in multiple cognitive domains and in 31% of patients with amnestic MCI [20].

In our study, in AD patients we found significant correlations between the results of DSC-MRI and FDG-PET in almost all the evaluated locations, apart from the right parietal cortex, while in the MCI group there was only a single correlation within the left PCG. The single correlation in the case of MCI was probably due to the small sample of subjects. After combing AD and MCI subjects in one group, significant correlations between DSC-MR perfusion and FDG-PET studies were revealed in all evaluated locations. The strongest correlations were revealed within temporal (r = 0.55–0.6) and PCG (r = 0.53–0.63) regions followed by parietal (r = 0.45–0.46) cortices, which are the regions of the most pronounced and typical changes in the course of AD degeneration. To our knowledge in the literature there are only two reports comparing DSC-MRI with FDG-PET in AD and MCI. In the first paper by Gonzales et al. the authors performed their study only on 10 patients with dementia (6 with AD) using visual evaluation of rCBV and brain glucose metabolism maps [15]. They compared the results within 8 brain layers and demonstrated a significant correlation (r = 0.62) at the levels of the upper and supraventricular layers. The mean correlation from all layers was r = 0.53, with the temporal area and the posterior fossa showing the weakest correlations (r = 0.24–0.33), which was explained by artifacts related to vessel pulsation. Our results do not fully agree with these findings, but it has to be stressed that our analyzes were conducted on a larger number of subjects and were based on parametrical values of rCBV and glucose metabolism, and thus seem to be more accurate than a visual assessment. In the second report, Zimny et al. showed a statistically significant correlation (r = 0.44) of rCBV measurements and FDG-PET results in PCG [18]. The results of this report are partially consistent with our findings in the left PCG (r = 0.4). However, the authors did not compare other regions of the brain and did not separate PCG into right and left regions.

To compare DSC-MR perfusion and FDG-PET results, we evaluated sensitivity and specificity and the accuracy of these two studies in distinguishing AD and MCI from healthy controls. We found a very similar high accuracy of DSC-MR perfusion and FDG-PET in distinguishing AD from the control group (0.98 and 0.97, respectively), and markedly higher accuracy of FDG-PET than DSC-MR perfusion in the differentiation of MCI from the control group (0.96 and 0.68–0.77, respectively). When distinguishing AD from MCI, both methods showed intermediate accuracy around 0.84 for MR and 0.81 for PET studies. It has to be stressed that though there are many reports in the literature showing the results of sensitivity, specificity and accuracy of DSC-MRI or FDG-PET in the diagnosis of AD or MCI, none of them were performed on the same groups of patients [12, 13, 16, 17, 20, 34–37].

There are several reports evaluating MR perfusion in the differentiation of AD from CG based on temporo-parietal areas and the results are slightly worse than in our study. For example, Harris et al. defined sensitivity as 0.95 in moderately affected patients with AD and 0.88 in mild cases of AD, whereas specificity as 0.96 [13]. In turn, Bozzao et al. in distinguishing AD from CG, achieved sensitivity of 0.91 and specificity of 0.87, while Maas et al. achieved 0.8 and 0.88 for sensitivity and specificity, respectively [12, 16]. On the other hand, Zimny et al. in the regions of PCG alone showed the accuracy of AD diagnosis as 0.87 [17], so lower than in our study (accuracy 1.0). Our results of FDG-PET in differentiating AD from CG (sensitivity 0.97, specificity 1.0, accuracy 0.97) are similar to other publications by Gambir et al. (sensitivity of 0.9–0.96, specificity of 0.67–0.97 and accuracy of 0.89), Mosconi et al. (sensitivity of 0.99, specificity of 0.98, accuracy of 0.98) or Gupta et al. (sensitivity of 0.9, specificity of 0.9 and accuracy of 0.92) and much higher compared to other studies reporting their sensitivity, specificity and accuracy results below 0.9 [20, 34–37]. It should be emphasized that in MCI subjects cognitive functions are impaired to an intermediate degree between proper aging and dementia, and there are so-called overlap periods, so distinguishing AD from MCI is a more difficult task than AD from CG [2]. To our knowledge, there are no reports in the literature in which authors could provide the accuracy values of MR perfusion in differentiating AD from MCI. In the differentiation of AD from MCI using the FDG-PET method, our results are similar to the literature. Gupta et al. when distinguishing AD from converting MCI assessed sensitivity, specificity and accuracy as 0.67, 0.88, and 0.81, respectively, (in our study 0.82, 0.77, and 0.81, respectively) [35]. According to De Santi et al. it is best to differentiate AD from MCI based on results of glucose metabolism in the temporal lobes, which is consistent with the results of our study, where accuracy from this cortical location was greater (0.9–0.92) than in other regions [38]. Regarding the differentiation of MCI from CG using DSC-MRI, our study showed better results  compared to several previous reports, for example by Zimny et al. who, based on evaluation of PCG, determined sensitivity, specificity and accuracy as 0.72, 0.8 and 0.7, respectively, and in the next study accuracy as 0.67 [17, 18]. In the differentiation of MCI from CG using the FDG-PET method, our results are similar to the literature [20, 22, 35]. For example, Gupta et al. in analyzing MCI converting to AD from CG, showed the sensitivity, specificity and accuracy as 0.98, 1.0, and 0.8, respectively, (in our study 0.95, 1.0, and 0.95, respectively).

In the last part of our study we evaluated correlations between the results of DSC-MRI or FDG-PET studies and the results of the MMSE test. In AD, statistically significant correlations were found with the results of DSC-MR perfusion from the left parietal and left temporal lobes, while in MCI in PCG and both parietal cortices. In the FDG-PET study, statistically significant correlations with MMSE were found only in AD patients with the results from the left frontal cortex. However, it should be emphasized that after putting together all AD and MCI subjects in one bigger group, a statistically significant correlation between MR perfusion or FDG-PET results and MMSE test was found in all the examined locations. Summarizing, it should be stated that in a larger group the results of these correlations are very similar for DSC-MR perfusion and FDG-PET. In the literature the results of correlation of MMSE test with DSC-MRI are ambiguous. Some authors showed no correlation of rCBV parameter with the MMSE test in AD or MCI patients [13, 31, 32] and several other authors found such correlations [16, 17]. The lack of correlation of psychological tests in separate groups of AD and MCI with FDG-PET results is in contradiction with several literature reports [37, 39–41].

Recently, more reports have focused on the comparison of FDG-PET with a non-contrast MR perfusion technique such as ASL. Fällmar et al. demonstrated a positive predictive value of ASL MR in AD and FTLD patients using visually analyzed perfusion maps and high specificity (0.84) of diagnoses, despite lower sensitivity (0.53) compared to FDG-PET (0.96) [42]. Similarly, Musiek et al. demonstrated, using visual inspection of perfusion and glucose metabolism maps, that both methods showed alterations in parieto-temporal areas, while the FDG-PET examination also depicted hypometabolism within the frontal lobes [43]. Johnson et al. comparing ASL MR and FDG-PET techniques in the AD group, showed that in both techniques the lower parts of the parietal lobes, PCG, superior and middle frontal gyrus were involved [44]. On the other hand, in the MCI group Johnson et al. showed a reduction in perfusion in the lower part of the right parietal lobe, which was slightly consistent with the pattern of glucose hypometabolism [44]. Riederer et al. also using the ASL MR method in MCI, showed no statistically significant differences in ASL perfusion rCBF parameter between aMCI and CG, contrary to FDG-PET studies, which showed hypometabolism on both sides of inferior parietal, superior temporal, right prefrontal dorsolateral cortex, precuneus, PCG and MTL [45]. All the above studies were performed using only visual inspection of ASL MRI and FDG-PET maps. Despite a growing interest in ASL perfusion due to the lack of contrast material needed during the examination, this MR method has several drawbacks. One of them is a prolonged acquisition time, which makes ASL impossible to be used in non-cooperative patients (e.g., those with advanced dementia). Other disadvantages are the necessity of three Tesla MR scanners to obtain reliable data, which are not widely available and a low signal-to-noise ratio (SNR).

The important advantage of the FDG-PET study is that its results may be partially evaluated based only on visual inspection, which is possible thanks to existing software such as CORTEX ID, which calculates the glucose metabolism normalized to the cerebellum and the z-score in relation to the database of healthy people, and automatically generates color-coded 3D maps of the cerebral cortex. There is no such software to post-process MR perfusion studies, which makes it impossible to visually assess the degree of hypoperfusion based on raw CBV maps. Assessment of DSC-MR perfusion results requires manual ROI placement and calculations of CBV values. Absolute CBV values cannot be evaluated, since they are dependent on several factors, such as blood hemodynamics or capillary permeability. This is why the relative value of CBV (compared to the cerebellar CBV) was used.

There are a few limitations of our study. Firstly, manual determination of ROIs is somewhat subjective and makes the method operator-dependent. Secondly, rather small groups of subjects may have had an impact on some results. We assessed more significant correlations after combining patients in a larger group of AD and MCI subjects. Another drawback is the cross-sectional character of the study. We have not evaluated longitudinal results regarding follow-up studies of aMCI subjects and the rate of their progression to dementia. It would be very interesting to check if DSC-MR perfusion has a similar strength as FDG-PET in predicting such a conversion.

## Conclusions

In our study, we proved that aMCI and AD patients show very similar patterns of hypoperfusion in DSC-MR and glucose hypometabolism in FDG-PET with a high rate of significant correlations between these two techniques. FDG-PET seems to be a better method in diagnosis of MCI, while DSC-MR perfusion was found to be more accurate in diagnosis of AD.

We believe that DSC-MR may be a good alternative to FDG-PET studies in patients with dementia. FDG-PET studies are still not widely available and very expensive, while MR examination is a routine study in the work-up of patients with dementia or MCI. A standard MR examination may be easily extended with DSC perfusion, which is a fast and fairly easy sequence to be performed. We believe that the development of dedicated software for DSC-MR perfusion post-processing could further facilitate its use in clinical practice.

## Data Availability

The datasets generated and analysed during the current study are available from the corresponding author on reasonable request.

## Abbreviations

AD: Alzheimer’s disease
aMCI: Amnestic mild cognitive impairment
ASL-MRI: Arterial spin labelling MRI
CBV: Cerebral blood volume
CG: Control group
CT: Computer tomography
DS-MRI: Dynamic susceptibility contrast enhanced MRI
FDG-PET: Fluorodeoxyglucose positron emission tomography
MMSE: Mini-Mental State Examination
MRI: Magnetic resonance imaging
PCG: Posterior cingulate gyrus
rCBV: Relative CBV
ROC: Receiver-operating characteristic
ROI: Regions of interest
SNR: Signal-to-noise ratio
